# Primary Progressive Aphasia in Italian and English: A Cross-Linguistic Cohort Study

**DOI:** 10.1212/WNL.0000000000210058

**Published:** 2024-11-21

**Authors:** Salvatore Mazzeo, Chris J.D. Hardy, Jessica Jiang, Carmen Morinelli, Valentina Moschini, Ella Brooks, Jeremy C.S. Johnson, Anthipa Chokesuwattanaskul, Giulia Giacomucci, Anna Volkmer, Jonathan D. Rohrer, Assunta Ingannato, Silvia Bagnoli, Sonia Padiglioni, Benedetta Nacmias, Sandro Sorbi, Valentina Bessi, Jason D. Warren

**Affiliations:** From the Dementia Research Centre (S.M., C.J.D.H., J.J., E.B., J.C.S.J., A.C., J.D.R., J.D.W.), Department of Neurodegenerative Disease, UCL Queen Square Institute of Neurology, University College London, United Kingdom; Research and Innovation Centre for Dementia-CRIDEM (S.M., C.M., V.M., S.P., S.S., V.B.), Azienda Ospedaliero-Universitaria Careggi, Florence; Vita-Salute San Raffaele University (S.M.), Milan; IRCCS Policlinico San Donato (S.M.), San Donato Milanese, Italy; Division of Neurology (A.C.), Department of Internal Medicine, King Chulalongkorn Memorial Hospital, Thai Red Cross Society; Cognitive Clinical and Computational Neuroscience Research Unit (A.C.), Faculty of Medicine, Chulalongkorn University, Bangkok, Thailand; University of Florence (G.G.), Italy; Department of Psychology & Language Sciences (A.V.), University College London, United Kingdom; Department of Neuroscience, Psychology, Drug Research and Child Health (A.I., S.B., B.N., S.S.), University of Florence, Azienda Ospedaliera-Universitaria Careggi; and IRCCS Fondazione Don Carlo Gnocchi (B.N., S.S., V.B.), Florence, Italy.

## Abstract

**Background and Objectives:**

Current formulations of primary progressive aphasia (PPA) derive largely from English-speaking patients. We hypothesized that language-specific characteristics influence PPA phenotypes in 2 contrasting languages: Italian and English.

**Methods:**

We undertook a retrospective, cross-sectional, observational comparison of 2 patient cohorts representing all major PPA syndromes, in London and Florence. Neuropsychological scores in a range of linguistic and general cognitive domains were normalized to native speaker controls and dichotomized as impaired/unimpaired. Proportions were compared using χ^2^ tests and adjusted for symptom duration and severity of cognitive impairment using logistic regression.

**Results:**

The cohorts comprised 106 (48.1% female) Italian speakers (14 nonfluent/agrammatic [nfvPPA], 20 semantic [svPPA], 41 logopenic variant [lvPPA], 31 mixed PPA [mPPA]) and 166 (45.2% female) English speakers (70 nfvPPA, 45 svPPA, 42 lvPPA, 9 mPPA). Comparing cohorts, the English cohort was younger (mean 62.7 [SD = 8.4] vs 65.9 [7.8] years; *p* = 0.003, Cohen *d* = 0.39), with longer symptom duration (4.6 [4.3] vs 3.1 [2.5] years; *p* = 0.048, *d* = 1.08), a higher proportion of nfvPPA cases (42% vs 13%, χ^2^ = 25.4, *p* < 0.001), and lower proportions of lvPPA (25% vs 38%, χ^2^ = 5.46, *p* = 0.019) and mPPA (5% vs 29%, χ^2^ = 29.3, *p* < 0.001). English-speaking nfvPPA patients had less frequent expressive agrammatism (46% vs 93%, *p* = 0.015, odds ratio [OR] 16.05, 95% CI 1.70–151.13) but more frequently impaired single-word comprehension (60% vs 8%, *p* = 0.013, OR 0.06, 95% CI 0.00–0.56). English svPPA patients had more frequent surface dyslexia (68% vs 30%, *p* = 0.046, OR 0.24, 95% CI 0.06–0.97) and dysgraphia (38% vs 10%, *p* = 0.021, OR 0.09, 95% CI 0.01–0.70) while English lvPPA patients had more frequently impaired single-word comprehension (89% vs 29%, *p* < 0.001, OR 0.05, 95% CI 0.01–0.28), word repetition (61% vs 26%, *p* = 0.020, OR 0.24, 95% CI 0.07–0.80), nonword repetition (78% vs 30%, *p* = 0.010, OR 0.18, 95% CI 0.05–0.06), nonverbal working memory (69% vs 36%, *p* = 0.005, OR 0.10, 95% CI 0.02–0.51), and visuomotor function (89% vs 25%, *p* < 0.001, OR 0.02, 95% CI 0.01–0.20).

**Discussion:**

Language-specific characteristics influenced PPA phenotypes, with more frequent expressive agrammatism in Italian (reflecting its morphologic complexity), more frequently impaired word processing in English (reflecting its articulatory, acoustic, and orthographic complexity), and increased prevalence of mPPA in Italian. These findings have implications for PPA diagnosis and management. Limitations of test heterogeneity and cohort size should be addressed in future, prospective, multicenter initiatives using cross-linguistic tools.

## Introduction

Primary progressive aphasia (PPA) denotes a diverse group of neurodegenerative disorders led by insidious deterioration of language skills.^1^ Current consensus diagnostic criteria for PPA recognize 3 canonical syndromic variants based on specific profiles of linguistic impairments^2^: nonfluent/agrammatic variant PPA (nfvPPA), semantic variant PPA (svPPA), and logopenic variant PPA (lvPPA). Diagnostic formulations and our wider understanding of PPA have largely been shaped by studies involving native English speakers.^1,2^ This is a serious issue: neurodegenerative pathologies targeting brain language networks are unlikely a priori to manifest uniformly across languages, given that languages vary widely in their linguistic, articulatory, acoustic, and orthographic characteristics and have diverse and far-reaching cultural associations. Studies of PPA syndromes in non–English-speaking patients have begun to identify language-specific signatures.^3-6^ To achieve accurate syndromic diagnosis, characterize clinical features fully, design relevant management approaches, and achieve inclusive recruitment to clinical trials, we require much more information about how PPA manifests in languages other than English. Only by studying PPA across languages can we assess commonalities and divergences, identify language-specific deficits and management needs, improve the reach of diagnostic tools, and evaluate the applicability of consensus diagnostic criteria, to decide how these might be more inclusively adapted. However, direct comparisons between PPA syndromes in different languages remain sparse, and we lack detailed cross-linguistic profiles covering the PPA spectrum.

Italian and English present contrasting linguistic features that may inform this issue. These 2 languages differ in characteristics such as morphologic typology, word order, and orthographic depth: Whereas English is an analytic language, with few inflections, strict word order, and “deep” orthography (i.e., it contains many orthographically irregular words), Italian is a synthetic language, highly inflected with flexible word order and “shallow” orthography. Further differences extend to the acoustic and articulatory properties of individual speech sounds: For example, English has a greater range of consonantal and vowel phonemes than Italian.^7^ Some evidence suggests that these language differences may be linked to contrasting phenotypes in Italian-speaking vs English-speaking patients with PPA and poststroke aphasia, based on the relative complexity (and likely neural computational demands) of processing particular linguistic features in each language. For example, the frequently inconsistent grapheme-phoneme correspondences of English predispose to the development of surface dyslexia; in Italian, this is much less frequently reported and chiefly manifests as errors of stress assignment.^6,8^ One comparative study in patients with nfvPPA revealed more impaired motor speech production in English speakers vs more severe agrammatism in Italian speakers.^5^ These findings align with evidence in poststroke aphasia^9,10^ and with the differential articulatory vs inflectional complexity of the 2 languages.

In this study, we undertook a detailed, retrospective comparison of neurolinguistic and general cognitive profiles in 2 large, well-characterized patient cohorts comprising Italian and English native speakers with all major syndromes of PPA. We used standardized, dichotomized (impaired/nonimpaired) test scores to compare cognitive profiles between cohorts. Based on the linguistic differences between Italian and English and previous evidence in patients with aphasia,^5-10^ we anticipated that the cognitive profiles of PPA syndromes would reflect the relative complexity of particular linguistic features in each language, interacting with the core deficits of canonical PPA phenotypes.^1^ We hypothesized that the complex inflections of Italian grammar would promote more frequent grammatical errors in Italian speakers, evident particularly in nfvPPA, whereas the greater phonetic and orthographic complexity of English would promote more frequent errors in auditory verbal tasks, evident particularly in lvPPA, and errors in reading and spelling tasks, evident particularly in svPPA. We further hypothesized a higher prevalence of mixed PPA (mPPA) phenotypes in the Italian cohort, given emerging evidence that such patients account for a substantial proportion of Italian-speaking PPA cases not fully characterized by the current diagnostic criteria.^11-14^

## Methods

### Characteristics of the Patient Cohorts

Italian-speaking patients (henceforth, the “Italian cohort”) were consecutively recruited and assessed at the Research and Innovation Centre for Dementia, Azienda Ospedaliero-Universitaria, Florence, between 2007 and 2022. English-speaking patients (henceforth, the “English cohort”) were consecutively recruited and assessed at the Dementia Research Centre, University College London. All were diagnosed according to current international consensus criteria,^2^ supported by a comprehensive clinical assessment and neuropsychological evaluation of language and other cognitive domains, and brain MRI and/or ^18^F-deoxyglucose-PET showing a compatible profile of regional atrophy and/or hypometabolism. Diagnoses of patients recruited before publication of the 2011 consensus criteria for PPA variants were retrospectively reviewed by 4 experienced cognitive neurologists (J.D.W. and J.D.R. for the English cohort, S.M. and V.B. for the Italian cohort), based on neuropsychological profiles and speech recordings. Patients were classified as having mPPA if they fulfilled the criteria for PPA but did not fulfill the criteria for a single canonical PPA variant.

English-speaking and Italian-speaking patients underwent research genetic screening for known pathogenetic variants in the *GRN*, *MAPT*, *C9orf72*, *APP*, *PSEN1*, and *PSEN2* genes, following previously described methods.^13,15^

All analyses comparing the 2 cohorts were undertaken retrospectively. We excluded patients with a history of significant intercurrent neurologic and/or systemic disease, prominent initial episodic memory, visuoperceptual or behavioral impairment, or severe language impairment precluding collection of speech samples of length and quality sufficient for meaningful analysis. None of the patients undergoing MRI showed a significant burden of cerebrovascular disease (modified Hachinski score ≤2).

### Standard Protocol Approvals, Registrations, and Patient Consents

All participants gave informed consent to take part in the relevant cohort study, and ethical approval was granted by the local institutional ethics committee (the University College London-National Hospital for Neurology and Neurosurgery Joint Research Ethics Committee or the Institutional Review Board of Careggi University Hospital, Florence, Italy, reference 15691oss), in accordance with Declaration of Helsinki guidelines.

### Language and General Cognitive Tests and Standardization Procedures

We selected tests to represent a range of linguistic and nonlinguistic cognitive domains from the neuropsychological batteries used at the Italian and English study centers to assess patients with acquired language disorders,^11,16^ summarized in Table 1.

**Table 1:** Neuropsychological Tests Used to Compare English and Italian Progressive Aphasia Cohorts

Cognitive domain assessed	Cohort	Test	Brief description	Max score
Overall cognitive performance	BOTH	Mini-Mental State Examination	30 questions probing several cognitive domains, weighted toward verbal responses	30
Language				
Phonemic fluency	BOTH	Phonemic fluency task	Subject asked to list as many words beginning (with letter F in English and with letters F, A, and S in Italian) as they can in 60 s	NA
Category fluency	BOTH	Category fluency task	Subject asked to list as many kinds of animals (in English) and animals, fruits, and car brands (in Italian) as they can in 60 s	NA
Naming	ENG	30-item Boston Naming Test	30 line drawings representing nouns of graded frequency ranging from “bed” to “protractor”	30
	ITA	SAND	14 black and white drawings including 7 living and 7 nonliving items. All items had low values of spoken frequency	14
Single-word comprehension	ENG	British Picture Vocabulary Scale	150 graded frequency words (spoken and visually presented) to be matched with pictures, 4 alternative forced choice on each trial	150
	ITA	SAND	12 spoken words to be matched with pictures, 4 alternative forced choice on each trial	12
Word repetition	ENG	Word repetition	45 words (15 mono/bi/tri-syllables) spoken by examiner, repeated by subject	45
	ITA	SAND	6 words (2 short-length words [5 phonemes] and 4 long words [between 8 and 10 phonemes]). Spoken by examiner, repeated by subject	6
Nonword repetition	ENG	Nonword repetition	20 nonwords (14 mono-/3 bi-/3 trisyllables) spoken by examiner, repeated by subject	20
	ITA	SAND	4 polysyllabic nonwords spoken by examiner, repeated by subject	4
Sentence repetition	ENG	Graded difficulty sentence repetition	10 graded length sentences spoken by examiner, repeated by subject	10
	ITA	SAND	6 sentences graded in length/grammatical complexity (3 predictable and 3 unpredictable, including 1 short [5 words] and 2 long [13 words] sentences) spoken by examiner, repeated by subject	6
Sentence comprehension	ENG	PALPA-55	24 spoken sentences to be matched with pictures, 3 alternative forced choice on each trial	24
	ITA	SAND	8 spoken sentences to be matched with pictures, 2 alternative forced choice on each trial	8
Expressive agrammatism	ENG	Written sentence production	Subject asked to produce 5 written sentences each incorporating 1 of 5 words nominated by examiner; scored as grammatically correct/agrammatic by neuropsychologist	5
	ITA	SAND	Propositional writing task (subject asked to describe how they brush their teeth); scored as grammatically correct/agrammatic by neuropsychologist	NA
Reading	ENG	National Adult Reading Test	50 irregularly spelled words read aloud by subject	50
	ITA	SAND	16 words (5 irregularly spelled^a^) read aloud by subject	16
Spelling	ENG	Written sentence production	Subject asked to produce 5 written sentences each incorporating 1 of 5 words nominated by examiner; scored as containing spelling errors or free of spelling errors by neuropsychologist	30
	ITA	SAND	Propositional writing task (subject asked to describe how they brush their teeth); scored as containing spelling errors or free of spelling errors by neuropsychologist	NA
Nonlinguistic cognitive functions				
Verbal working memory^b^	BOTH	Digit span: forward and reverse	Sequence of numbers (between 1 and 9) spoken by examiner, repeated in same order or reverse order by subject; increasing sequence length on successive trials	8
Nonverbal working memory^b^	BOTH	Corsi blocks: visuospatial span forward and reverse	Sequence of blocks in spatial array touched by examiner, repeated in same order or reverse order by subject; increasing sequence length on successive trials	9
Episodic memory: verbal	ENG	Recognition Memory Test: words	50 words spoken by examiner and encoded by subject, who is then required to identify the previously presented words from 50 visually presented word pairs after a delay	50
	ITA	Rey Auditory Verbal Learning Test	15 words spoken by examiner, for recall by subject after 15 min	15
Episodic memory: nonverbal	ENG	Recognition Memory Test: faces	50 pictures of faces encoded by subject, who is then required to identify the previously presented faces from 50 face pairs after a delay	50
	ITA	Rey-Osterrieth Complex Figure Recall	Subject asked to reproduce a drawing of a complex figure, 10 min after copying the same figure	36
Visuomotor function	BOTH	Trail Making Test: part A	Subject asked to draw a line connecting a sequence of 25 numbers, in order as quickly as possible	NA
Executive function	BOTH	Trail Making Test: part B	Subject asked to draw a line connecting a sequence of letters alternating with numbers, in order as quickly as possible	NA

Abbreviations: F = female; M = male; PALPA-55 = Psycholinguistic Assessments of Language Processing in Aphasia subtest 55; SAND = Screening for Aphasia in NeuroDegeneration.

The table summarizes the neuropsychological tests administered to the Italian (ITA) and English (ENG) cohorts in this study. Norms for each test were generated from healthy control cohorts of cognitively normal native speakers. Control cohort demographic parameters for tests used were as follows: English cohort (all tests), n = 104 (59 female), mean (SD) age 66.91 (6.73) years, education 15.81 (2.70) years; Italian cohort (varied by test), Mini-Mental State Examination, n = 1019 (769 female), age 75.4 (5.4) years, education 5.2 (2.5) years; phonemic fluency, n = 340 (163 female), age 53.1 (18.0) years, education 10.2 (4.3) years; category fluency, n = 320 (150 female), mean age 47.5 years, mean education 10.1 years (SD values not available); digit span and Corsi blocks, n = 362 (175 female), age 54.2 (24.1) years, 11.3 (4.6) years; Rey Auditory Verbal Learning Test, n = 340 (163 female), age 53.1 (18.0) years, education 10.2 (4.3) years; Rey-Osterrieth Complex Figure Recall, n = 280 (140 female), age 53.9 (19.8) years, education 11.1 (4.8) years; Trail Making Test, n = 287 (154 female), age 42.2 (15.4) years, education 11.4 (4.7) years; other tests, n = 134 (78 female), age 63.3 (11.2) years, education 11.0 (4.9) years. Normative data were not available for the tests of grammar and spelling used here. Further details are in the eMethods.

a Presence/absence of surface dyslexia in the Italian cohort recorded on reading aloud irregular words.

b Total score used.

To ensure comparability of the neuropsychological protocols between the English and Italian cohorts, tests were selected if (1) the same test had been used to assess a particular cognitive domain in both cohorts or (2) equivalent tests assessing the same cognitive domain were available for both cohorts and performance on those tests could be standardized based on healthy control norms for each language. We assessed the following linguistic domains: picture naming, phonemic fluency, category fluency, single-word comprehension, sentence comprehension, word repetition, nonword repetition, sentence repetition, sentence production (expressive grammatical construction), reading, and spelling. Assessment of literacy skills presents a particular challenge when comparing languages that vary in orthographic depth: In this study, reading of orthographically irregular words was assessed in both English and Italian, and any regularization errors due to sounding these words phonetically (surface dyslexia) were recorded, while the spelling tests assessed both orthographically regular and irregular words (further details in Table 1). Nonlinguistic cognitive domains comprised verbal working memory, nonverbal working memory, verbal and spatial episodic memory, and visuomotor and executive functions.

### Statistical Analysis

All statistical comparisons between cohorts were performed using IBM SPSS Statistics software version 25 (SPSS Inc., Chicago, IL) and the computing environment R 4.2.3 (R Foundation for Statistical Computing, Vienna, Austria, 2013). Distribution normality for variables was assessed using the Shapiro-Wilk test. We extracted descriptive statistics to examine the central tendency and variability of the data using means and SDs for continuous variables and frequencies or percentages for categorical variables. To compare cohorts and syndromic groups, we used *t* tests for normally distributed variables and χ^2^ tests for categorical data. To estimate the influence of differential disease severity, comparisons of cohort neuropsychological profiles were additionally adjusted for symptom duration and overall level of cognitive impairment (Mini-Mental State Examination [MMSE] score) using logistic regression. We calculated effect sizes using Cohen *d* for normally distributed numeric measures and Cramer *V* for categorical data.

To compare the cohorts on neuropsychological measures, raw neuropsychological test scores were transformed categorically as follows. For the English cohort, raw scores on each test were first converted to *w*-scores^17^ based on mean and SD scores for that test in a cohort of 104 healthy British controls, adjusted for age and years of education using previously described procedures.^18,19^ For the Italian cohort, raw scores on each test were first adjusted for age and years of education based on published normative data for the Italian population^20-26^ and then transformed to *z*-scores based on published means and SD for that test (see Table 1 for details of the norming cohorts). Based on standardized values, all test scores were next dichotomized as “impaired” (*z*-score (for the Italian cohort) or *w*-score (for the English cohort) >1.65, equivalent to fifth percentile performance) or “not impaired.” Standardized scores could not be calculated for agrammatism and spelling because of a lack of normative data for the tests used to assess these domains; performance in these domains was accordingly scored as impaired (some errors) or not impaired (no errors). The Italian and English cohorts were compared across cognitive domains based on the proportion of cases in each cohort showing a deficit for each cognitive domain.

A formal power calculation was performed to assess the adequacy of group sizes to demonstrate effects of interest (details in eMethods).^27^

### Data Availability

Anonymized data not published in this article will be made available by request from any qualified investigator.

## Results

### Summary of the Patient Cohorts

General demographic and clinical characteristics of the Italian and English PPA cohorts are summarized in Table 2.

**Table 2:** General Characteristics of Italian and English Primary Progressive Aphasia Cohorts

Characteristic	All syndromes		nfvPPA		svPPA		lvPPA		mPPA	
Native language	English	Italian	English	Italian	English	Italian	English	Italian	English	Italian
Total, n (%)	166	106	**70 (42.2)** ^**a**^	**14 (13.2)** ^**a**^	45 (27.1)	20 (18.9)	**42 (25.3)** ^**b**^	**41 (38.7)** ^**b**^	**9 (5.4)** ^**c**^	**31 (29.3)** ^**c**^
Female, n (%)	75 (45.2)	51 (48.1)	39 (55.7)	6 (42.9)	19 (42.2)	9 (45.0)	13 (30.9)	20 (48.8)	4 (44.4)	16 (51.6)
Handedness (R:L)^n^	115:16	66:3	44:7	10:0	33:5	9:1	31:4	25:1	9:0	31:0
Education (y)	**14.3 (2.9)** ^**d**^	**11.2 (5.3)** ^**d**^	**13.9 (2.8)** ^**e**^	**7.6 (3.5)** ^**e**^	**14.2 (13.5)** ^**f**^	**11.7 (4.5)** ^**f**^	15.2 (3.1)	13.3 (6)	**14.9 (2.2)** ^**g**^	**10.3 (4.9)** ^**g**^
Age at onset (y)	**62.7 (8.4)** ^**h**^	**65.9 (7.8)** ^**h**^	65.5 (8.6)	67.1 (7.3)	58.9 (7.9)	60.3 (7.8)	61.7 (6.9)	64.6 (7.7)	**64.0 (8.3)** ^**i**^	**70.6 (5.3)** ^**i**^
Age at assessment (y)	67.5 (8.5)	69.5 (7.6)	69.9 (8.8)	69.7 (7.7)	64.3 (8.3)	64.8 (9.2)	67.0 (7.1)	68.2 (7.4)	67.2 (8.7)	73.6 (4.6)
Symptom duration (y)	**4.6 (4.3)** ^**j**^	**3.1 (2.5)** ^**j**^	**4.3 (2.5)** ^**k**^	**2.6 (1.6)** ^**k**^	**5.4 (2.5)** ^**l**^	**3.6 (2.3)** ^**l**^	**4.7 (2.1)** ^**m**^	**3.1 (3.2)** ^**m**^	3.2 (1.2)	3.1 (2)
MMSE score	20.9 (7.6)	20.2 (5.7)	21.8 (7.6)	20.5 (6.0)	21.2 (8.6)	22.0 (5.6)	18.6 (6.7)	19.4 (6.2)	24.2 (3.83)	19.8 (5.74)
Pathogenetic variants^o^	7 *GRN* 2 *C9orf72* 1 *MAPT* 2 *PSEN1*	2 *GRN* 1 *MAPT* 1 *PSEN2*	4 *GRN* 2 *C9orf72* 1 *PSEN1*	1 *GRN*	1 *MAPT*	—	1 *GRN* 1 *PSEN1*	1 *MAPT* 1 *PSEN2* 1 *GRN*	2 *GRN*	—

Abbreviations: lvPPA = patient groups with logopenic variant primary progressive aphasia; MMSE = Mini-Mental State Examination; mPPA = patient groups with mixed/atypical primary progressive aphasia; nfvPPA = patient groups with nonfluent/agrammatic primary progressive aphasia; svPPA = patient groups with semantic variant primary progressive aphasia.

The table summarizes general demographic and clinical characteristics of the combined Italian and English primary progressive aphasia cohorts and major syndromic groups within each cohort. Values quoted are means (with SDs) for continuous variables and frequencies (percentages) for dichotomous variables. Groups were compared between cohorts using *t* tests or (for categorical data) χ^2^ tests; effect sizes were assessed using Cohen *d* for continuous measures and Cramer *V* for categorical data. Statistically significant cohort group differences (*p* < 0.05) are indicated in bold. Parameter values were as follows: ^a^χ^2^ = 25.42, *p* < 0.001, *V* = 0.31; ^b^χ^2^ = 5.46, *p* = 0.019, *V* = 0.14; ^c^χ^2^ = 29.27, *p* < 0.001, *V* = 0.33; ^d^*p* < 0.001, *d* = 0.76; ^e^*p* < 0.001, *d* = 2.15; ^f^*p* = 0.033, *d* = 0.70; ^g^*p* = 0.001, *d* = 0.99; ^h^*p* = 0.003, *d* = 0.39; ^i^*p* = 0.048, *d* = 1.08; ^j^*p* < 0.001, *d* = 0.63; ^k^*p* = 0.004, *d* = 0.68; ^l^*p* = 0.008, *d* = 0.75; ^m^*p* = 0.011, *d* = 0.61. Statistical significance accepted at *p* < 0.05.

n Data on handedness available for 131 English-speaking and 69 Italian-speaking patients.

o Pathogenetic variants identified in genes listed.

The English cohort comprised 166 consecutive patients with PPA (70 [42%] nfvPPA, 45 [27%] svPPA, 42 [25%] lvPPA, 9 [5%] mPPA). The Italian cohort comprised 106 consecutive patients with PPA (14 [13%] nfvPPA, 20 [19%] svPPA, 41 [39%] lvPPA, 31 [29%] mPPA). In both the English and Italian cohorts, female and male patients were approximately equally represented (45.2% vs 48.1% female patients, respectively). Across cohorts, only 1 patient was bilingual (syndromic diagnosis, mPPA; languages Italian and French). A total of 125 English-speaking and 94 Italian-speaking patients underwent research genetic screening for known pathogenetic variants in *GRN*, *MAPT*, *C9orf72*, *APP*, *PSEN1*, and *PSEN2* genes. The 104 age-matched healthy British controls had a mean age of 66.9 years [SD = 6.7] and mean educational attainment of 15.8 years [SD = 2.7]. The formal power calculation indicated that we had sufficient group sizes to detect a relevant clinical effect both for the cohorts as a whole and for the smallest syndromic group (mPPA, see eMethods).

### Comparison of General Cohort Characteristics

The Italian and English cohorts showed no significant differences in sex distribution, handedness, age at assessment, or overall cognitive severity (MMSE score). English speakers with PPA had a significantly younger age at onset (62.7 [8.4] vs 65.9 [7.8]), longer mean symptom duration (4.6 [4.3] vs 3.10 [2.5]), and more years of education (14.3 [2.9] vs 11.2 [5.3]) than Italian speakers. Within syndromic groups, similarly directed significant cohort differences were also observed for symptom duration (in nfvPPA, svPPA, and lvPPA), education (in nfvPPA, svPPA, and mPPA), and age at onset (in mPPA) (Table 2).

The English and Italian cohorts showed distinct profiles of syndromic representation. The English cohort had a higher proportion of nfvPPA cases (42.2% vs 13.2%, χ^2^ = 25.4, *p* < 0.001, *V* = 0.31) and lower proportion of lvPPA (25.3% vs 38.7%, χ^2^ = 5.46, *p* = 0.019, *V* = 0.14) and mPPA (5.4% vs 29.3%, χ^2^ = 29.3, *p* < 0.001, *V* = 0.33) cases than the Italian cohort.

Genetic analysis revealed a low frequency of pathogenetic variants across both cohorts. Pathogenetic variants in *GRN* were most often represented (9 cases in total; 4 English nfvPPA, 1 English lvPPA, 2 English mPPA, 1 Italian nfvPPA, 1 Italian lvPPA), followed by *MAPT* (2 cases; 1 English svPPA, 1 Italian lvPPA), *C9orf72* (2 cases, both English nfvPPA), *PSEN1* (2 cases; 1 English nfvPPA, 1 English lvPPA) and *PSEN2* (1 Italian lvPPA). Overall, 12 of 125 (9.6%) of the English cohort and 4 of 94 (4.2%) of the Italian cohort were found to harbor pathogenetic variants; these proportions did not differ significantly between the cohorts (*p* = 0.132).

### Comparison of Neuropsychological Characteristics Between Cohorts

**Figure 1:**
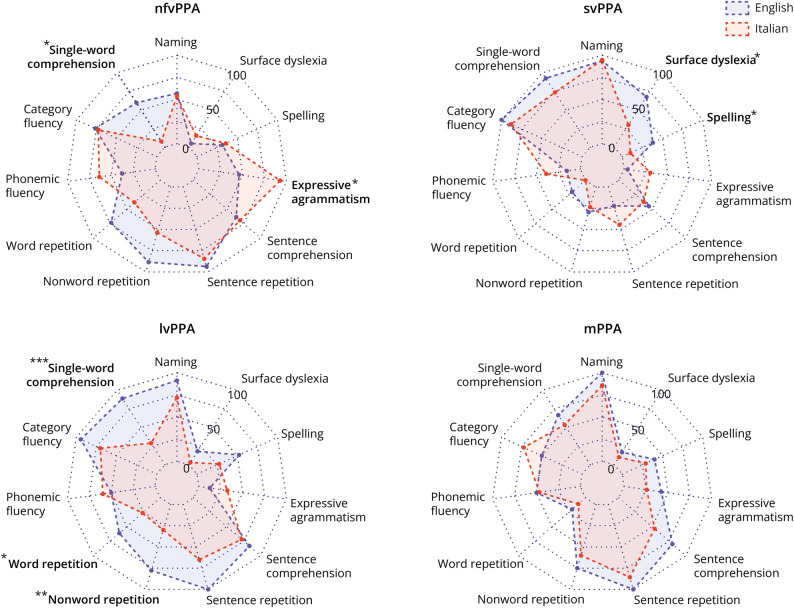
Profiles of Neurolinguistic Impairment in the Italian and English Primary Progressive Aphasia Cohorts For each major syndromic category, the radar plot represents the proportion of patients showing a deficit in each cognitive domain after *z*-score/*w*-score transformation (see text and Table 3). Statistically significant differences between cohorts after adjustment for MMSE score and symptom duration, coded as follows: *p < 0.05; **p < 0.01; ***p < 0.001, are indicated in bold. lvPPA = logopenic variant primary progressive aphasia; MMSE = Mini-Mental State Examination; mPPA = mixed/atypical primary progressive aphasia; nfvPPA = nonfluent/agrammatic primary progressive aphasia; svPPA = semantic variant primary progressive aphasia.

**Figure 2:**
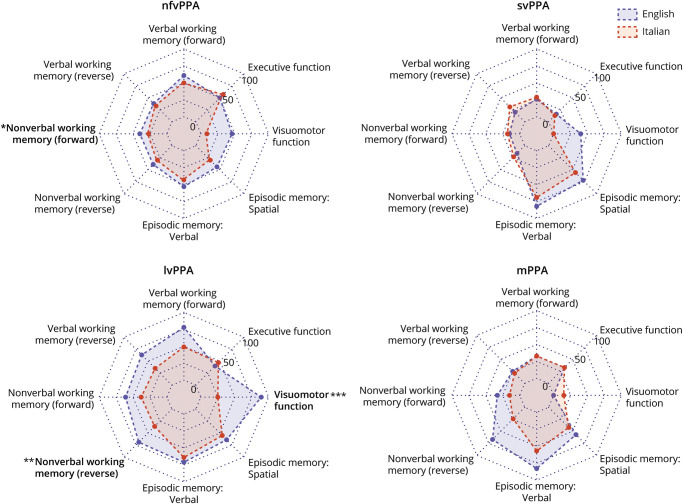
Profiles of Nonlinguistic General Cognitive Impairment in the Italian and English Primary Progressive Aphasia Cohorts For each major syndromic category, the radar plot represents the proportion of patients showing a deficit in each cognitive domain after *z*-score/*w*-score transformation (see text and Table 3). Statistically significant differences between cohorts after adjustment for MMSE score and symptom duration, coded as follows: *p < 0.05; **p < 0.01; ***p < 0.001, are indicated in bold. lvPPA = logopenic variant primary progressive aphasia; MMSE = Mini-Mental State Examination; mPPA = mixed/atypical primary progressive aphasia; nfvPPA = nonfluent/agrammatic primary progressive aphasia; svPPA = semantic variant primary progressive aphasia.

Neuropsychological data for the 2 cohorts (English vs Italian) are compared in Table 3 and Figures 1 and 2.

**Table 3:** Comparison of the Italian and English Primary Progressive Aphasia Cohorts on Neuropsychological Measures

Native language	nfvPPA				svPPA				lvPPA				mPPA			
	English (n = 70)	Italian (n = 14)	Comparison		English (n = 45)	Italian (n = 20)	Comparison		English (n = 42)	Italian (n = 41)	Comparison		English (n = 9)	Italian (n = 31)	Comparison	
			*p*_unadj_	*p*_adj_			*p*_unadj_	*p*_adj_			*p*_unadj_	*p*_adj_			*p*_unadj_	*p*_adj_
Linguistic functions																
Phonemic fluency	38%	64%	0.182	0.372	15%	39%	0.155	0.689	50%	59%	0.507	0.688	50%	47%	0.900	0.245
Category fluency	76%	73%	0.823	0.146	100%	88%	0.185	0.997	**94%**	**70%**	**0.042**	0.149	50%	72%	0.361	0.409
Naming	57%	54%	0.850	0.945	94%	95%	0.885	0.775	**91%**	**72%**	**0.036**	0.093	100%	86%	0.367	0.996
Single-word comprehension	***60%***	***8%***	***<0.001***	***0.013***	**93%**	**74%**	**0.044**	0.113	***89%***	***29%***	***<0.001***	***<0.001***	67%	54%	0.490	0.433
Word repetition	**73%**	**38%**	**0.020**	0.093	**20%**	**0%**	**0.016**	0.994	***61%***	***26%***	***0.003***	***0.020***	20%	11%	0.558	0.497
Nonword repetition	**89%**	**54%**	**0.008**	0.093	30%	24%	0.658	0.643	***78%***	***30%***	***<0.001***	***0.010***	75%	60%	0.562	0.941
Sentence repetition	94%	85%	0.442	0.425	22%	44%	0.260	0.691	100%	65%	0.058	0.995	100%	86%	0.483	0.996
Sentence comprehension	64%	69%	0.710	0.308	45%	37%	0.583	0.323	83%	72%	0.260	0.240	80%	54%	0.271	0.250
Expressive agrammatism	***46%***	***93%***	***0.008***	***0.015***	**4%**	**30%**	**0.018**	0.069	13%	33%	0.127	0.262	43%	26%	0.511	0.779
Reading^a^	5%	15%	0.208	0.265	***68%***	***30%***	***0.005***	***0.046***	**18%**	**3%**	**0.042**	0.174	17%	10%	0.635	0.397
Spelling	31%	36%	0.773	0.751	***38%***	***10%***	***0.024***	***0.021***	52%	28%	0.050	0.092	40%	29%	0.621	0.357
Other cognitive functions																
Verbal working memory (forward)	61%	50%	0.331	0.851	26%	29%	0.801	0.759	**78%**	**49%**	**0.009**	0.422	33%	33%	1.00	0.377
Verbal working memory (reverse)	38%	33%	0.787	0.998	20%	31%	0.395	0.073	**63%**	**35%**	**0.022**	0.354	25%	21%	0.830	0.496
Nonverbal working memory (forward)	***40%***	***27%***	***0.434***	***0.041***	15%	18%	0.820	0.618	61%	38%	0.067	0.201	33%	15%	0.414	0.064
Nonverbal working memory (reverse)	39%	30%	0.610	0.249	16%	23%	0.586	0.617	***69%***	***36%***	***0.010***	***0.005***	67%	24%	0.122	0.332
Episodic memory: verbal	53%	43%	0.613	0.210	82%	69%	0.279	0.060	71%	64%	0.583	0.254	83%	57%	0.228	0.192
Episodic memory: nonverbal	44%	30%	0.396	0.602	72%	56%	0.257	0.199	64%	55%	0.451	0.622	57%	42%	0.484	0.116
Visuomotor function	**46%**	**9%**	**0.034**	0.063	**40%**	**0%**	**0.003**	0.996	***89%***	***25%***	***<0.001***	***<0.001***	0%	15%	0.474	0.996
Executive function	50%	57%	0.748	0.795	16%	13%	0.841	0.754	40%	47%	0.705	0.516	33%	33%	1.00	0.305

Abbreviations: lvPPA = patient groups with logopenic variant primary progressive aphasia; mPPA = patient groups with mixed/atypical primary progressive aphasia; nfvPPA = patient groups with nonfluent/agrammatic primary progressive aphasia; svPPA = patient groups with semantic variant primary progressive aphasia.

Percentages indicate the proportions of patients in each cohort with impairment in the language and nonlinguistic cognitive domains listed in the first column (derived from age-adjusted and years of education–adjusted scores); details of neuropsychological tests administered are in Table 1. For cohort comparisons, *p*_unadj_ indicates the level of significance computed using χ^2^ tests; *p*_adj_ indicates the significance level of the cohort (English minus Italian) difference variable in the logistic regression model including symptom duration and MMSE score as covariates (see text). Proportions that differed significantly between groups are indicated in bold; those that differed after adjustment are given in bold italics. Statistical significance was accepted at *p* < 0.05 for all tests.

a Deficit here signifies surface dyslexia.

#### nfvPPA

The English nfvPPA group showed significantly more frequent impairment in single-word comprehension (60% vs 8%, χ^2^ = 11.4, *p* < 0.001, *V* = 0.40), nonword repetition (89% vs 54%, χ^2^ = 6.9, *p* = 0.008, *V* = 0.38), word repetition (73% vs 38%, χ^2^ = 5.4, *p* = 0.020, *V* = 0.31), and visuomotor function (46% vs 9%, χ^2^ = 4.53, *p* = 0.034, *V* = 0.36). The Italian nfvPPA group had a significantly higher frequency of expressive agrammatism (93% vs 46%, χ^2^ = 9.20, *p* = 0.002, *V* = 0.43). After adjusting for symptom duration and MMSE score, differences in single-word comprehension (*p* = 0.013, odds ratio [OR] 0.06, 95% CI 0.00–0.56) and expressive agrammatism (*p* = 0.015, OR 16.05, 95% CI 1.70–151.13) remained significant, whereas nonverbal working memory impairment was found to be significantly more frequent in English-speaking patients (*p* = 0.041, OR 0.04, 95% CI 0.00–0.88).

#### svPPA

The English svPPA group showed significantly more frequent impairment in visuomotor function (40% vs 0%, χ^2^ = 9.12, *p* = 0.003, *V* = 0.49), reading irregular words (surface dyslexia; 68% vs 30%, χ^2^ = 7.9, *p* = 0.005, *V* = 0.38), word repetition (20% vs 0%, χ^2^ = 5.9, *p* = 0.016, *V* = 0.34), spelling (38% vs 10%, χ^2^ = 5.1, *p* = 0.024, *V* = 0.38), and single-word comprehension (93% vs 74%, χ^2^ = 4.06, *p* = 0.044, *V* = 0.26). The Italian svPPA group had a significantly higher frequency of expressive agrammatism (30% vs 4%, χ^2^ = 5.6, *p* = 0.018, *V* = 0.34). After adjusting for symptom duration and MMSE score, differences in dysgraphia (*p* = 0.021, OR 0.09, 95% CI 0.01–0.70) and surface dyslexia (*p* = 0.046, OR 0.24, 95% CI 0.06–0.97) remained significant.

#### lvPPA

The English lvPPA group was significantly more frequently impaired in visuomotor function (89% vs 25%, χ^2^ = 18.8, *p* < 0.001, *V* = 0.61), single-word comprehension (89% vs 29%, χ^2^ = 27.3, *p* < 0.001, *V* = 0.60), nonword repetition (78% vs 30%, χ^2^ = 14.7, *p* < 0.001, *V* = 0.47), word repetition (61% vs 26%, χ^2^ = 9.12, *p* = 0.003, *V* = 0.35), verbal working memory (digit span forward: 78% vs 49%, χ^2^ = 6.8, *p* = 0.009, *V* = 0.30; digit span reverse: 63% vs 35%, χ^2^ = 5.2, *p* = 0.022, *V* = 0.27), nonverbal working memory (visuospatial span reverse: 69% vs 36%, χ^2^ = 6.5, *p* = 0.010, *V* = 0.33), naming (91% vs 72%, χ^2^ = 4.4, *p* = 0.036, *V* = 0.25), category fluency (94% vs 70%, χ^2^ = 4.2, *p* = 0.042, *V* = 0.27), and reading irregular words (18% vs 3%, χ^2^ = 4.1, *p* = 0.042, *V* = 0.24). The Italian lvPPA group was not significantly more impaired than the English lvPPA group in any neuropsychological domain. After adjusting for symptom duration and MMSE score, differences in visuomotor function (*p* < 0.001, OR 0.02, 95% CI 0.01–0.20), single-word comprehension (*p* < 0.001, OR 0.05, 95% CI 0.01–0.28), nonverbal working memory (*p* = 0.005, OR 0.10, 95% CI 0.02–0.51), nonword repetition (*p* = 0.010, OR 0.18, 95% CI 0.05–0.06), and word repetition (*p* = 0.020, OR 0.24, 95% CI 0.07–0.80) remained significant.

#### mPPA

The English and Italian mPPA groups did not differ significantly on any of the assessed neuropsychological characteristics at the prescribed threshold.

## Discussion

In this study, we have shown that the relative frequencies and cognitive profiles of PPA syndromes differ for native speakers of 2 major world languages, Italian and English. In line with population demographic data^28^ and previous work in PPA,^5^ the English cohort on average had a longer education, were younger at symptom onset, and had a longer symptom duration compared with the Italian cohort. Accordingly, we adjusted for each of these factors in analysis and focus on these adjusted findings below. Across syndromes, the English cohort had a higher frequency of specific language deficits than the Italian cohort, although overall cognitive severity was similar in both cohorts. Pathogenic genetic variants were infrequent, occurring in fewer than 10% of cases in both cohorts, and therefore unlikely to have driven phenotypic cohort differences. Although all cases in both cohorts were diagnosed based on current international consensus criteria,^2^ nfvPPA was significantly more frequently diagnosed in the English cohort, whereas lvPPA and mPPA were more frequently diagnosed in the Italian cohort. The relative syndromic proportions in the English PPA cohort here are similar to those reported for previous large British and North American English-speaking PPA cohorts.^29,30^ These proportions contrast with the present Italian cohort, particularly in the relative frequencies of nfvPPA, lvPPA, and mPPA, whereas a previous large pan-European study reported nfvPPA and mPPA syndromic frequencies intermediate between the present English and Italian cohorts.^31^

For nfvPPA, expressive agrammatism was significantly more frequent in the Italian cohort and impaired single-word comprehension in the English cohort; for svPPA, reading (surface dyslexia) and spelling deficits were more frequent in the English cohort; and for lvPPA, single-word comprehension and repetition deficits were more frequent in the English cohort. These syndromic differences were echoed across the PPA cohorts as a whole: Expressive agrammatism in the Italian cohort and single-word comprehension, repetition, and surface dyslexic deficits in the English cohort were more frequent overall. These profiles are consistent with the grammatical, phonologic, and orthographic differences between the 2 languages,^7^ although the cohorts also differed to a lesser extent in their profiles of nonlinguistic cognitive impairment. Our use of dichotomized “counts” of impairment frequency (based on standardized scores) to characterize language profiles in the 2 cohorts raises certain caveats. This methodology precludes comparison of severity differences within cognitive domains. Relatedly, referencing patient performance to language-specific normal control data leaves open the possibility that the tests used to assess a particular cognitive domain may have differed in intrinsic difficulty between English and Italian (i.e., the tests used to assess that domain may have been more likely to reveal an impairment in one language than the other). Particular cognitive domains should not, therefore, be considered more relevant than others when assessing PPA in individual speakers of different languages. Rather, our findings suggest overall performance patterns that might guide assessment and indicate a need for caution when interpreting performance particularly in languages other than English.

The diagnosis of nfvPPA rests chiefly on the presence of speech apraxia and expressive agrammatism,^2^ and the relative prominence of these features in English and Italian speakers with nfvPPA has previously been shown to differ in line with the present findings.^5^ Although we did not have a direct measure of speech sound production errors here, speech apraxia can show language-specific signatures^32^ and may be more likely to manifest in English than Italian. Whereas English is characterized by complex consonantal clusters that place heavy demands on articulation and a diverse range of vowel sounds, Italian has a simpler and more fluid syllabic structure.^7,33^ Whereas English tends to express grammatical relationships through word order and auxiliary verbs, Italian uses a rich set of morphologic features that may impose a higher computational load on failing combinatorial mechanisms.^34^

The greater proportion of single-word comprehension deficits in English-speaking patients with nfvPPA and lvPPA is unlikely to reflect the assessment methods used (because a 4-alternative, forced-choice, spoken word-picture matching task was used in both cohorts^26,35^). A potential neurolinguistic basis for this discrepancy is suggested by the aligned cohort differences in real-word and nonword repetition performance (which attained significance for lvPPA). Relatively greater impairment of auditory phonologic decoding in English-speaking patients might lead both to more frequent disruption of auditory working memory and reduced comprehension of spoken words. The greater acoustic complexity of English relative to Italian may be relevant here,^7^ and impaired auditory function at or before the stage of phonologic decoding has been documented in English speakers with lvPPA and nfvPPA.^36-38^ It is noteworthy that impaired single-word comprehension has previously been reported in Dutch-speaking patients with nfvPPA^39^: Similarly to English, Dutch contains frequent, acoustically complex consonant clusters. Furthermore, the discrepancy in spoken word comprehension between cohorts in our study contrasts with their very similar naming performance (Table 3), arguing for an auditory rather than primary semantic basis. It is further possible that any phonologic input deficit is amplified by concomitantly more marked deficits of phonologic production in English-speaking patients with nfvPPA and lvPPA, through impaired lexical predictive processing.^40,41^

In the case of svPPA, the higher frequency of surface dyslexia and dysgraphia in English speakers compared with Italian speakers can be analogously understood as a consequence of the substantially greater prevalence and variety of orthographically irregular words in English.^42^ However, the reading tests used in the 2 cohorts differed in their proportions of irregularly spelled words, whereas the spelling tests used different tasks (Table 1): Caution is therefore required when interpreting these deficits.

Cohort differences for nonverbal functions were more restricted than for language functions and may at least in part reflect the longer mean illness duration in English-speaking patients. Though we adjusted for overall severity of cognitive impairment using MMSE score, this is unlikely to fully account for differences in the complex sequence of nonverbal cognitive changes that accompany evolving PPA syndromes^43,44^; moreover, it is weighted toward verbal capacities. There are currently no ideal measures of disease severity covering the entire PPA spectrum: This is an important direction for future work.

The frequency of mPPA was disproportionately higher in the Italian cohort compared with the English cohort. Considering the English and Italian PPA cohorts together, this study demonstrates that the development of mPPA is not driven by disease duration or severity; indeed, it may manifest on average earlier than canonical PPA syndromes (Table 2). An increased proportion of “unclassified” Italian mPPA cases would follow if the consensus diagnostic criteria,^2^ developed for English-speaking patients, cannot capture the full phenotypic spectrum of Italian PPA. However, the neurolinguistic profiles of mPPA cases in the English and Italian cohorts were strikingly similar (Figure 1), arguing for underlying mechanisms that are at least partly common to both language groups. Certain features of the mPPA profile here—in particular the preponderance of naming and sentence repetition deficits—would tend to align it with lvPPA, as previously noted for Italian speakers with mPPA^13^ and in accordance with emerging formulations of lvPPA as a multidimensional entity that is loosely demarcated from other PPA syndromes and Alzheimer disease.^11,45^ However, recent work in Italian patients with mPPA has shown that a substantial proportion do not have Alzheimer biomarkers,^12^ supporting a distinct pathophysiologic process that remains to be defined. Considered together, the present evidence argues for a reappraisal of the current diagnostic criteria to achieve a more inclusive coverage of the PPA phenotypic spectrum and suggests this may be even more pressing for PPA in languages other than English.

This study has several limitations that should help direct future work. Most fundamentally, the English and Italian cohorts did not complete the same neurolinguistic test battery: Although we standardized and dichotomized raw scores to mitigate this, intrinsic test difficulty cannot readily be equated between languages, and the healthy control reference populations also differed so that standardized scores may not be equivalent between cohorts. Moreover, the sampling of language and other cognitive domains was incomplete (e.g., we had no comparison measure of speech apraxia and only assessed a relatively restricted range of nonverbal cognitive skills). It is debatable whether any conventional neuropsychological battery could be entirely fairly translated between languages; emerging alternative approaches, for example, computer-assisted analysis of speech and natural language use, may in future go some way toward achieving a universal, cross-linguistic assessment framework for PPA.^46^ Within the cognitive domains sampled, neuropsychological test scores did not align perfectly with the clinical syndromic diagnosis (Table 3); more lenient criteria for impairment might have improved alignment but would have tended to make true differences between the cohorts more difficult to detect. Furthermore, different reference healthy control groups were used for each cohort, and neither control group was study-specific.

The syndromic groups in our study were relatively small, drawn from only 2 centers, and assessed retrospectively, with notable differences in sample size between the English and Italian cohorts, particularly for the nfvPPA and mPPA groups. While these discrepancies in part reflect natural variation in syndrome frequency between the cohorts, the small number of Italian patients with nfvPPA and English patients with mPPA means that inferences about cross-linguistic syndromic differences must be provisional awaiting corroboration with larger samples. Given the relatively small cohort sizes here, we elected not to apply a multiple comparisons correction in the statistical analysis, in order not to overlook true cohort differences. However, this also means that the hypothesis-driven language differences we have identified here require confirmation in studies with more robust statistical power. Future cross-linguistic studies should prospectively assess larger PPA cohorts in the context of a multicenter collaboration, comparing additional languages, and tracking the longitudinal evolution of deficits. Studies of this kind are already underway^15^ and may be particularly pertinent to rare syndromes such as those associated with genetic PPA.

Our findings suggest that specific language features may shape the prevalence and presentation of major PPA syndromes in Italian and English speakers, after adjusting for potentially confounding associations. Expressive agrammatism is more frequent in Italian (reflecting its morphologic complexity), and word processing is more frequently affected in English (reflecting its articulatory, acoustic and orthographic complexity), whereas Italian speakers are more likely to present with mPPA. These findings have important implications for PPA diagnosis, management, and clinical trial design, and indeed, potentially for the broader spectrum of communication dysfunction in neurodegenerative diseases such as Alzheimer disease with global reach. The consensus diagnostic criteria for PPA may not optimally capture the spectrum of PPA phenotypes in different languages: Improving diagnostic inclusivity will depend on large-scale, international collaborative initiatives potentially using new cross-linguistic tools^46,47^ and integrating neurolinguistics with measures of daily-life function and neural biomarkers. Ultimately, accurate diagnosis is required to direct management: improved recognition of language-specific PPA features would facilitate inclusive, bespoke treatments of individual patients with PPA, potentially guiding both nonpharmacological interventions such as speech and language therapy and the deployment of disease-modifying therapies.^47,48^ Furthermore, cross-linguistic tools will be essential for recruitment and assessment of outcomes in clinical trials of new therapies for PPA, in a new era of global equity.^47^

## Appendix Authors

**Table:**

Name	Location	Contribution
Salvatore Mazzeo, MD, PhD	Dementia Research Centre, Department of Neurodegenerative Disease, UCL Queen Square Institute of Neurology, University College London, United Kingdom; Research and Innovation Centre for Dementia-CRIDEM, Azienda Ospedaliero-Universitaria Careggi, Florence; Vita-Salute San Raffaele University, Milan; IRCCS Policlinico San Donato, San Donato Milanese, Italy	Drafting/revision of the manuscript for content, including medical writing for content; major role in the acquisition of data; study concept or design; analysis or interpretation of data
Chris J.D. Hardy, PhD	Dementia Research Centre, Department of Neurodegenerative Disease, UCL Queen Square Institute of Neurology, University College London, United Kingdom	Drafting/revision of the manuscript for content, including medical writing for content; major role in the acquisition of data; study concept or design; analysis or interpretation of data
Jessica Jiang, PhD	Dementia Research Centre, Department of Neurodegenerative Disease, UCL Queen Square Institute of Neurology, University College London, United Kingdom	Drafting/revision of the manuscript for content, including medical writing for content; major role in the acquisition of data
Carmen Morinelli, PsyD	Research and Innovation Centre for Dementia-CRIDEM, Azienda Ospedaliero-Universitaria Careggi, Florence, Italy	Drafting/revision of the manuscript for content, including medical writing for content; major role in the acquisition of data
Valentina Moschini, PsyD	Research and Innovation Centre for Dementia-CRIDEM, Azienda Ospedaliero-Universitaria Careggi, Florence, Italy	Drafting/revision of the manuscript for content, including medical writing for content; major role in the acquisition of data
Ella Brooks, MSc	Dementia Research Centre, Department of Neurodegenerative Disease, UCL Queen Square Institute of Neurology, University College London, United Kingdom	Drafting/revision of the manuscript for content, including medical writing for content
Jeremy C.S. Johnson, MD, PhD	Dementia Research Centre, Department of Neurodegenerative Disease, UCL Queen Square Institute of Neurology, University College London, United Kingdom	Drafting/revision of the manuscript for content, including medical writing for content; major role in the acquisition of data
Anthipa Chokesuwattanaskul, MD	Dementia Research Centre, Department of Neurodegenerative Disease, UCL Queen Square Institute of Neurology, University College London, United Kingdom; Division of Neurology, Department of Internal Medicine, King Chulalongkorn Memorial Hospital, Thai Red Cross Society; Cognitive Clinical and Computational Neuroscience Research Unit, Faculty of Medicine, Chulalongkorn University, Bangkok, Thailand	Drafting/revision of the manuscript for content, including medical writing for content; major role in the acquisition of data
Giulia Giacomucci, MD	University of Florence, Italy	Major role in the acquisition of data
Anna Volkmer, PhD	Department of Psychology & Language Sciences, University College London, United Kingdom	Drafting/revision of the manuscript for content, including medical writing for content; major role in the acquisition of data
Jonathan D. Rohrer, PhD, FRCP	Dementia Research Centre, Department of Neurodegenerative Disease, UCL Queen Square Institute of Neurology, University College London, United Kingdom	Drafting/revision of the manuscript for content, including medical writing for content; major role in the acquisition of data
Assunta Ingannato, PhD	Department of Neuroscience, Psychology, Drug Research and Child Health, University of Florence, Azienda Ospedaliera-Universitaria Careggi, Italy	Drafting/revision of the manuscript for content, including medical writing for content; major role in the acquisition of data
Silvia Bagnoli, PhD	Department of Neuroscience, Psychology, Drug Research and Child Health, University of Florence, Azienda Ospedaliera-Universitaria Careggi, Italy	Drafting/revision of the manuscript for content, including medical writing for content; major role in the acquisition of data
Sonia Padiglioni, PsyD	Research and Innovation Centre for Dementia-CRIDEM, Azienda Ospedaliero-Universitaria Careggi, Florence, Italy	Drafting/revision of the manuscript for content, including medical writing for content; major role in the acquisition of data
Benedetta Nacmias, PhD	Department of Neuroscience, Psychology, Drug Research and Child Health, University of Florence, Azienda Ospedaliera-Universitaria Careggi; IRCCS Fondazione Don Carlo Gnocchi, Florence, Italy	Drafting/revision of the manuscript for content, including medical writing for content; major role in the acquisition of data
Sandro Sorbi, MD	Research and Innovation Centre for Dementia-CRIDEM, and Department of Neuroscience, Psychology, Drug Research and Child Health, University of Florence, Azienda Ospedaliera-Universitaria Careggi; IRCCS Fondazione Don Carlo Gnocchi, Florence, Italy	Drafting/revision of the manuscript for content, including medical writing for content; major role in the acquisition of data
Valentina Bessi, MD, PhD	Research and Innovation Centre for Dementia-CRIDEM, Azienda Ospedaliero-Universitaria Careggi; IRCCS Fondazione Don Carlo Gnocchi, Florence, Italy	Drafting/revision of the manuscript for content, including medical writing for content; major role in the acquisition of data; study concept or design
Jason D. Warren, PhD, FRACP	Dementia Research Centre, Department of Neurodegenerative Disease, UCL Queen Square Institute of Neurology, University College London, United Kingdom	Drafting/revision of the manuscript for content, including medical writing for content; major role in the acquisition of data; study concept or design; analysis or interpretation of data

## Glossary

lvPPA: logopenic variant PPA
MMSE: Mini-Mental State Examination
mPPA: mixed PPA
nfvPPA: nonfluent/agrammatic PPA
OR: odds ratio
PPA: primary progressive aphasia
svPPA: semantic variant PPA
